# A higher CD4/CD8 ratio correlates with an ultralow cell-associated HIV-1 DNA level in chronically infected patients on antiretroviral therapy: a case control study

**DOI:** 10.1186/s12879-017-2866-y

**Published:** 2017-12-15

**Authors:** Yongsong Yue, Nidan Wang, Yang Han, Ting Zhu, Jing Xie, Zhifeng Qiu, Xiaojing Song, Yanling Li, Jean-Pierre Routy, Jianhua Wang, Taisheng Li

**Affiliations:** 1 Department of Infectious Diseases, Peking Union Medical College Hospital, Chinese Academy of Medical Sciences & Peking Union Medical College, Beijing, 100730 China; 2 Clinical Immunology Center, Chinese Academy of Medical Sciences, Beijing, 100730 China; 3 Center for AIDS Research, Chinese Academy of Medical Sciences and Peking Union Medical College, Beijing, 100730 China; 4 Division of Hematology & Chronic Viral Illness Service, McGill University Health Centre, Quebec, Canada; 5 CAS Key Laboratory of Molecular Virology and Immunology, Institute Pasteur of Shanghai, Chinese Academy of Sciences, Shanghai, 200031 China; 6 University of Chinese Academy of Sciences, Beijing, China

**Keywords:** HIV-1, DNA reservoir, Antiretroviral therapy, CD8^+^ T-lymphocytes, CD4/CD8 ratio

## Abstract

**Background:**

The HIV-1 DNA reservoir is an important marker that reflects viro-immunological status and can be affected by multiple viral or cellular factors. Determining the potential factors associated with the size of the HIV-1 DNA reservoir benefits the surveillance of disease progression and antiretroviral treatments.

**Methods:**

We conducted a case control study to explore the factors that may affect the level of HIV-1 DNA. The level of HIV-1 total DNA in peripheral blood at 5 time points was quantified by quantitative PCR. Chronically HIV-1-infected patients whose cell-associated HIV-1 DNA levels were below the detection limit after receiving antiretroviral therapy (ART) for 96 weeks were identified (group 1), and patients who still had detectable levels of cell-associated HIV-1 DNA after ATR treatment were used as the control (group 2).

**Results:**

Twenty-one patients with ultralow levels of cell-associated HIV-1 DNA [<20 copies/10^6^ peripheral blood mononuclear cells (PBMCs)] presented with a lower CD8^+^ T-cell count (average: 511 ± 191 versus 715 ± 256 cells/μL, *p* = 0.013) and a higher CD4/CD8 ratio (average: 1.04 ± 0.37 versus 0.72 ± 0.32, respectively, *p* = 0.002) at week 96. In the multivariate analysis, patients with a higher CD4/CD8 ratio at week 96 were more likely to have levels of HIV-1 DNA below the detection limit (per 0.1 increase, OR = 1.29, 95% CI, 1.05–1.59, *p* = 0.017).

**Conclusion:**

After matching baseline HIV-1 DNA levels, a higher CD4/CD8 ratio at week 96 was the only factor associated with an ultralow level of HIV-1 DNA. The CD4/CD8 ratio can be used as an easy biomarker to help monitor patients on ART who will be selected to participate in eradication studies.

**Electronic supplementary material:**

The online version of this article (10.1186/s12879-017-2866-y) contains supplementary material, which is available to authorized users.

## Background

HIV-1 persistence in infected memory CD4^+^ T cells represents a major obstacle for eradication even after long-term antiretroviral treatment [1, 2]. Establishment of HIV-1 DNA reservoirs occurs days after HIV-1 infection and exists in different tissues, including lymph nodes and preferentially in memory CD4^+^ T cells [3–6]. The size of the HIV-1 DNA reservoir measured before antiretroviral therapy (ART) initiation is an important marker that reflects the viro-immunological status, is independently correlated with disease progression, and can be used to predict the timing of viral rebound after treatment interruption [7–10].

The level of peripheral blood mononuclear cell (PBMC)-associated HIV-1 DNA can be affected by multiple factors, including the plasma viral load, the peripheral CD4^+^ T-lymphocyte count, the timing of ART initiation and the duration of ART treatment as well as the immune activation status [11–17]. Early and prolonged treatment, in concert with optimal viral control and high CD4^+^ T-cell count, is associated with a lower level of HIV-1 DNA [18]. In rare cases, post-treatment controllers also reduce the level of HIV-1 DNA, as in the VISCONTI study [19].

Determining the potential viral or cellular factors that are associated with the size of the HIV-1 DNA reservoir benefits the surveillance of disease progression and antiretroviral treatments. In the current case control study, we identified 21 patients with chronic HIV-1 infection who had undetectable cell-associated HIV-1 DNA levels after 2 years of ART treatment. The 48 other patients, who still had detectable cell-associated HIV-1 DNA levels after ATR treatment, were used as the control. We focused on analyzing other factors associated with below-detection-level DNA by matching baseline HIV-1 DNA levels between the two groups and found that the CD4/CD8 ratio at week 96 was the only factor associated with an ultralow HIV-1 DNA level.

## Methods

### Subjects

Sixty-nine patients in this study were selected from two previous adult HIV-1 treatment cohorts [20, 21]: cohort 1, recruited between 2008 and 2010, consisting of patients with a CD4^+^ T-cell count <350 cells/μL and receiving an ART regimen of zidovudine/stavudine + lamividine + nevirapine (AZT/d4T + 3TC + NVP); and cohort 4, recruited between 2012 and 2014, comprising patients with a CD4^+^ T-cell count <500 cells/μL and receiving an ART regimen of tenofovir + lamividine + efavirenz (TDF + 3TC + EFV). Peripheral blood was collected for T-cell enumeration and plasma viral load detection, and the remainder from each visit was stored at −80 °C [pre-ART (week 0), 3 months (week 12), 6 months (week 24), 1 year (week 48), and 2 years after initiation ART (week 96)]. Demographic characteristics, CD4^+^ and CD8^+^ T-cell counts, HIV-1 viral subtype, and plasma HIV-1 RNA viral loads were extracted from the database.

HIV-1 DNA quantification was performed in 868 patients with available samples from these two cohorts. Among them, 651 patients achieved viral suppression (viral load <50 copies/mL) within 48 weeks of ART. Further, 21 patients with ultralow HIV-1 DNA levels (<20 copies/10^6^ PBMCs) at week 96 and strict viral suppression (HIV-1 RNA maintained <50 copies/mL after 48 weeks of ART without viral blip) were selected in group 1. To analyze other factors associated with ultralow HIV-1 DNA levels, patients with strict viral suppression but detectable HIV-1 DNA at week 96 were selected for the control group (group 2) by matching baseline HIV-1 DNA levels. According to the proportion of 1:2 (number of patients in group 1: group 2), 42 patients were tended to be randomly chosen from those with baseline HIV-1 DNA <2.4 log copies/10^6^ PBMCs (75 quantile of HIV-1 DNA in group 1). In fact, only 48 patients met that criterion, and the level of baseline HIV-1 DNA was balanced with group 1. Hence, those 48 patients were assigned to group 2.

All 69 patients were treatment-naïve when enrolled, achieved and maintained viral suppression (<50 copies/mL) without blip after 48 weeks of ART and were followed for 96 weeks. Among them, 45 (65%) patients achieved viral suppression at week 12, 18 (26%) patients at week 24, and 6 (9%) patients at week 48 on ART. During follow-up (96 weeks), their viral loads were always less than 50 copies/mL without blip.

### HIV DNA quantification

Total cellular DNA was extracted from 200 μL peripheral blood using Qiagen QIAsymphony DNA Mini Kits (QIAGEN, Valencia, CA), and HIV-1 DNA in the peripheral blood (mainly the white blood cells, WBCs) was amplified and quantified for LTR gene using a fluorescence-based, real-time SUPBIO HIV Quantitative Detection Kit (SUPBIO, Guangzhou, China). The quantification range of this assay was 20–5 × 10^6^ copies/10^6^ WBCs. The amount of HIV-1 DNA per 10^6^ PBMCs was calculated.

### Statistical analysis

Differences in continuous and categorical variables between the two groups were assessed using standard non-parametric tests and the chi-squared test, respectively. Undetectable HIV-1 RNA and DNA results were given the value of the detection limit (20 copies/mL for HIV-1 RNA and 20 copies/10^6^ PBMCs for HIV-1 DNA). Univariate and multivariate logistic regression were employed to identify predictors of below-detection-level total HIV-1 DNA. Factors with a *p* value <0.10 in the univariate logistic regression analysis were included in a multivariate logistic regression model, and all factors were subjected to backward-stepwise likelihood ratio (LR) regression with *p* < 0.20 as the exclusion criterion. Statistical analysis was performed using SPSS 22.0 (IBM Corporation, Armonk, New York, USA) and GraphPad Prism 6.0 (GraphPad Software, Inc. La Jolla, CA, USA). A *p* value <0.05 was considered statistically significant.

## Results

### Baseline characteristics of the patients

In the case control study, the 69 patients with successful treatment were selected from the cohorts and were stratified into two groups according to the level of cell-associated HIV-1 DNA after 96 weeks of ART. In group 1, the 21 patients had undetectable HIV-1 DNA (<20 copies/10^6^ PBMCs) after 96 weeks of ART, and in group 2 (control), the 48 patients still had detectable cell-associated HIV-1 DNA after ATR treatment [median log copies/10^6^ PBMCs, 1.91; inter-quartile range (IQR), 1.6–2.4, *p* < 0.001]. The other characteristics of the two groups, including sex, age, HIV-1 subtype, the antiretroviral regimens, pre-ART HIV-1 DNA and plasma RNA, pre-ART CD4^+^ and CD8^+^ T-cell counts, and nadir CD4^+^ T-cell count, were all matched (Table 1).

**Table 1:** Characteristics of the overall study population and stratification by HIV-1 DNA level at week 96

Variables	Total (*N* = 69)	Group 1 (*N* = 21)	Group 2 (*N* = 48)	*P* value
Sex				0.082
Male	43 (62.3%)	10 (47.6%)	33 (68.8%)	
Female	26 (37.7%)	11 (52.4%)	15 (31.2%)	
Age (years)	33 (27–39)	33 (29–41)	33 (27–38)	0.396
Time from diagnosis to ART (months)	7.7 (1.1–25.5)	6.0 (1.0–16.0)	9.0 (1.2–28.0)	0.556
HIV-1 subtype				0.422
AE	21 (30.4%)	5 (23.8%)	16 (33.3%)	
B/C/BC	19 (27.5%)	6 (28.6%)	13 (27.1%)	
Not known	29 (42.1%)	10 (47.6%)	19 (39.6%)	
ART therapy				0.332
AZT/d4T + 3TC + NVP	19 (27.5%)	7 (33.3%)	12 (25.0%)	
TDF + 3TC + EFV	50 (72.5%)	14 (66.7%)	36 (75.0%)	
Pre-ART HIV-1 DNA (log_10_ copies/10^6^ PBMCs), median (IQR),	2.0 (1.7–2.2)	1.8 (1.5–2.4)	2.0 (1.8–2.2)	0.379
Pre-ART plasma viral load (log_10_ copies/mL), median (IQR)	4.1 (3.6–4.5)	4.0 (3.6–4.4)	4.2 (3.7–4.6)	0.445
Pre-ART CD4^+^ T-cell count (cells/μL), median (IQR)	305 (256–385)	305 (274–412)	306 (232–378)	0.312
Nadir CD4^+^ T-cell count (cells/μL), median (IQR)	292 (245–357)	295 (271–374)	291 (224–350)	0.240
Pre-ART CD8^+^ T-cell count (cells/μL), median (IQR)	845 (614–1073)	859 (634–1114)	763 (607–1074)	0.907
Pre-ART CD4/CD8 ratio, median (IQR)	0.35 (0.27–0.51)	0.42 (0.29–0.51)	0.34 (0.24–0.49)	0.180
HIV-1 DNA after 2 years’ ART (log10 copies/10^6^ PBMCs), median (IQR)	1.7 (1.3–2.0)	1.3 (1.3–1.3)	1.9 (1.6–2.4)	<0.001

*ART* Antiretroviral therapy, *PBMCs* Peripheral blood mononuclear cells, *IQR* Inter-quartile range

The information of these 69 patients is summarized in Table 1. A total of 62.3% of patients were male. The median age was 33 years (IQR, 27–39). Among them, 21 patients were infected with the HIV-1 AE subtype and 19 patients with either the B, C, or BC subtype. All patients were diagnosed with HIV-1 and received ART during the chronic phase. The median time from diagnosis to ART was 7.7 months. The baseline virological and immunological characteristics of these patients were as follows: median pre-ART plasma viral load, 4.1 (IQR, 3.6–4.5) log copies/mL; median CD4^+^ T-cell count, 305 (IQR, 256–385) cells/μL; median nadir CD4^+^ T-cell count, 292 (IQR, 245–357) cells/μL; median CD8^+^ T-cell count, 845 (IQR, 614–1073) cells/μL; median CD4/CD8 ratio, 0.35 (IQR, 0.27–0.51); and median pre-ART HIV total DNA, 2.0 (IQR, 1.7–2.2) log copies/10^6^ PBMCs. Two types of antiretroviral regimens were used. Nineteen patients (27.5%) received AZT/d4T + 3TC + NVP, and 50 patients (72.5%) received TDF + 3TC + EFV.

### Temporal changes in the plasma viral load, CD4^+^ and CD8^+^ T-cell counts, CD4/CD8 ratio, and HIV-1 DNA level during ART

**Fig. 1:**
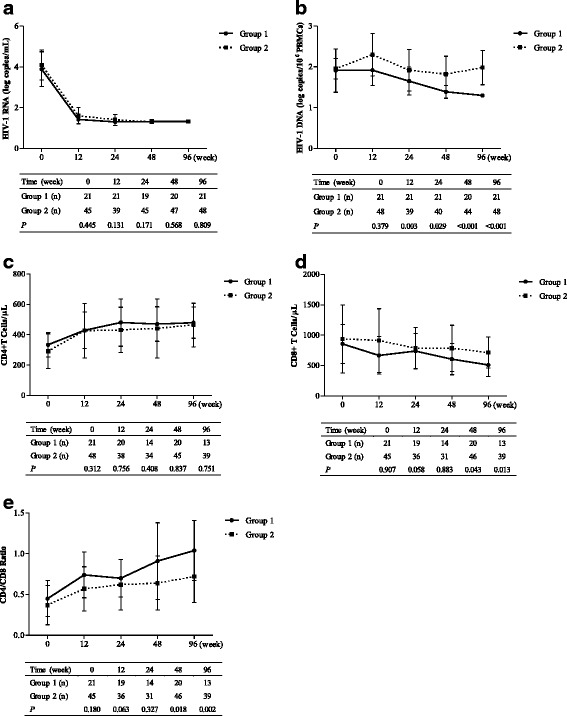
Dynamic changes to cellular and viral parameters in HIV-1-infected patients during ART treatment. In these retrospective studies, the time-course parameters, including the viral load (**a**), HIV-1 DNA level (**b**), and peripheral CD4^+^ (**c**) and CD8^+^ T-lymphocyte counts (**d**) from ATR-treated patients were recorded, and the CD4/CD8 ratio was calculated (**e**). *P* < 0.05 is considered significant

These 69 patients showed suppressed HIV-1 RNA production after 48 weeks of ART, and there was no obvious difference in the plasma viral load between the two groups at the evaluation time points (Fig. 1a). A significant difference in the cell-associated HIV-1 DNA level appeared at week 12 after initiating ART (*p* = 0.003), and the difference persisted at weeks 24 and 96 (*p* = 0.029 and *p* < 0.001, respectively, Fig. 1b). Upon ATR treatment, the CD4^+^ T-cell count showed a rapid recovery within 12 weeks, but no significant difference in the CD4^+^ T-cell count was observed between the two groups at any evaluated time points (Fig. 1c). Intriguingly, the number of CD8^+^ T cells in group 1 continued to decrease after week 24, resulting in a significantly lower CD8^+^ T-cell count at week 48 (*p* = 0.043) and week 96 (*p* = 0.013, Fig. 1d). Consequently, a higher CD4/CD8 ratio was observed at these two visits (*p* = 0.018 and *p* = 0.002, respectively, Fig. 1e).

### Higher CD4/CD8 ratio is associated with undetectable HIV-1 DNA at 96 weeks of ART

To identify which factors were associated with undetectable HIV-1 DNA levels at 96 weeks of ART in group 1, a binary logistic regression model was used for the analysis. Univariate logistic regression revealed that a lower CD8^+^ T-cell count and a higher CD4/CD8 ratio at week 96 were beneficial factors associated with undetectable HIV-1 DNA levels at 96 weeks of ART in group 1 (*p* = 0.018 and 0.014, respectively, Table 2). However, only the CD4/CD8 ratio at week 96 remained after multivariate logistic regression. Patients with a higher CD4/CD8 ratio were more likely to achieve ultralow HIV-1 DNA levels after 2 years of ART [per 0.1 increase, odds ratio (OR) = 1.29, 95% confidence interval (CI), 1.05–1.59, *p* = 0.017, Table 2].

**Table 2:** Factors associated with an ultralow HIV-1 DNA level (<20 copies/10^6^ PBMCs) at week 96

Variables		Univariate			Multivariate	
	OR	95% CI	*P*	OR	95% CI	*P*
Sex			0.100			0.092
Male	1.00			1.00		
Female	2.42	(0.85–6.93)		3.40	(0.82–14.15)	
Age (years)	1.01	(0.96–1.07)	0.633			
Time from diagnosis to ART (months)	0.98	(0.94–1.01)	0.183			
Transmission route			0.857			
Homosexual	1.00					
Heterosexual	1.36	(0.44–4.27)				
Others	1.42	(0.20–9.82)				
HIV-1 subtype			0.717			
AE	1.00					
B/C/BC	1.48	(0.37–5.96)	0.584			
Others	1.68	(0.48–5.95)	0.418			
ART therapy			0.477			
AZT/d4T + 3TC + NVP	1.00					
TDF + 3TC + EFV	0.67	(0.22–2.04)				
Plasma viral load at enrollment (log copies/mL)	0.71	(0.36–1.41)	0.330			
HIV-1 DNA at enrollment (log copies/10^6^ PBMCs)	0.72	(0.17–3.11)	0.656			
CD4^+^ T-cell count at enrollment (cells/μL)	1.00	(1.00–1.01)	0.134			
Nadir CD4^+^ T-cell count (cells/μL)	1.00	(1.00–1.01)	0.129			
CD8^+^ T-cell count at enrollment (cells/μL)	1.00	(1.00–1.00)	0.528			
CD4/CD8 ratio at enrollment	1.19	(0.95–1.48)	0.124			
Plasma viral load at week 96			0.808			
Undetectable	1.00					
Viremia	0.75	(0.07–7.66)				
CD4^+^ T-cell count at week 96 (cells/μL)	1.00	(1.00–1.00)	0.709			
CD8^+^ T-cell count at week 96 (cells/μL)	0.99	(0.99–0.99)^a^	0.018	1.00	(0.99–1.00)	0.462
CD4/CD8 ratio at week 96 (per 0.1 increase)	1.28	(1.05–1.55)	0.014	1.29	(1.05–1.59)	0.017

*OR* Odds ratio, *CI* confidence interval, *ART* Antiretroviral therapy, *PBMCs* Peripheral blood mononuclear cells

^a^, 0.993~0.999

In our study, all participants were virally suppressed (HIV-1 RNA < 50 copies/mL) at week 96. Detectable low-level viremia at week 96 (20–50 copies/mL) was seen in 4 patients. To eliminate the effect of residual viremia on the level of HIV-1 DNA, we analyzed only patients with undetectable HIV-1 RNA. As before, the CD4/CD8 ratio at week 96 was the only factor associated with undetectable HIV-1 DNA at week 96 (OR = 1.26, 95% CI, 1.04–1.53, *p* = 0.021, Additional file 1: Table S1).

## Discussion

This was a case control study of chronically HIV-1-infected patients receiving ART for 2 years. Twenty-one patients had an HIV-1 DNA level lower than the detection limit at week 96. After matching the two groups for baseline HIV-1 DNA levels, the CD4/CD8 ratio at week 96 was the only factor that was associated with an ultralow HIV-1 DNA level.

The most striking discovery of this study was that we found 21 patients who achieved an undetectable level of the HIV-1 DNA. The most important feature of this group of 21 patients was their relatively lower baseline HIV-1 DNA level (median, 1.8 log copies/10^6^ PBMCs, Table 1), which was significantly lower than that of the screened population (median 3.05 log copies/10^6^ PBMCs; data not shown). A previous study showed that the HIV-1 DNA level following ART was strongly associated with the pre-ART HIV-1 DNA level [14]. We focused on analyzing other factors associated with below-detection-level DNA by matching the baseline HIV-1 DNA level in the two groups.

During successful antiretroviral treatment, we observed no difference in viral inhibition or CD4^+^ T-cell recovery between the two groups. Nevertheless, we found significant differences in the CD8^+^ T-cell count and CD4/CD8 ratio between the two groups at weeks 48 and 96. CD8^+^ T cells, which become elevated soon after infection and seldom normalize despite effective ART, are required for maintaining viral suppression and are independently associated with non-AIDS-related clinical events [22, 23]. Our study showed that a reduction of the CD8^+^ T-cell count was associated with an HIV-1 reservoir below the detection level. Moreover, the CD4/CD8 ratio at week 96 was the only factor related to an ultralow HIV-1 DNA outcome. Chun et al. first revealed an inverse correlation between the CD4/CD8 ratio and frequency of CD4^+^ T cells carrying HIV-1 proviral DNA in infected individuals receiving ART [24]. Later, in 2012, this result was confirmed by Boulassel et al., who showed that the date of infection, duration of ART, and patient age did not influence the DNA level, in contrast to nadir CD4^+^ T-cell count and CD4/CD8 ratio at baseline [12]. The nadir CD4^+^ T-cell count, however, did not influence the DNA outcome in our study. Recently, Rajesh and his colleagues applied a more ultrasensitive HIV-1 DNA detection assay and demonstrated that lower CD4/CD8 ratio was associated with persistently higher HIV-1 DNA during ART [25]. Our findings confirm and further extend these previous results.

For a long time, studies have placed intense focus on the inverse correlation between CD4^+^ T cells and the DNA reservoir [11–13]. In our study, we found no difference in the recovery of CD4^+^ T cells or the DNA reservoir. However, we found a sustained decrease in the number of CD8^+^ T cells in group 1 during ART, resulting in a continuous increase in the CD4/CD8 ratio, which has been considered a marker for immune activation and a parameter for disease progression for many years [26–28]. Patients with a lower CD4/CD8 ratio after long-term ART have a higher risk of non-AIDS-related morbidities and mortalities [28–31]. In fact, the relationship between the size of the HIV-1 DNA reservoir and level of immune activation has been observed in several studies, showing a positive association between the integrated HIV-1 DNA load and frequency of activated CD4^+^ or CD8^+^ T cells (HLA-DR^+^, CD38^+^, PD-1^+^) during consistently suppressive therapy [16, 17, 32, 33]. The current opinion is that ART suppresses HIV-1 replication, resulting in progressive CD4^+^ T-cell recovery but incomplete restoration of the CD4/CD8 ratio. A low CD4/CD8 ratio indicates a high extent of immune activation and enhanced homeostatic proliferation of HIV-1-infected CD4^+^ T cells, resulting in the persistence and perpetuation of the DNA reservoir [34, 35].

There were some limitations to our study. In addition to the small sample size, the duration of ART in our study was not sufficient to fully understand the long-term effects of ART (or the CD4/CD8 ratio) on the HIV-1 DNA reservoir. However, the reservoir stayed stable after 1 to 2 years of ART. Furthermore, immune activation data such as the dynamic changes in HLA-DR and CD38 markers expressed on the surface of CD4^+^ and CD8^+^ T cells were lacking, and this information may help provide insight into the relationship between the CD4/CD8 ratio and HIV-1 DNA reservoir. In addition, we detected HIV-1 DNA only in peripheral blood, whereas larger HIV-1 reservoirs exist in gut-associated lymph tissues and lymph nodes [36, 37]. Whether an ultralow level of HIV-1 DNA can also be found in these structures remains unknown.

## Conclusions

In conclusion, our study is the first to observe a below-detection-level HIV-1 DNA reservoir in a small group of chronically HIV-1-infected patients after receiving ART for 2 years. After matching the HIV-1 DNA level of our two groups at baseline, CD4/CD8 ratio at week 96 was the only factor associated with an ultralow HIV-1 DNA outcome. Considering that a lower total HIV-1 DNA level and better immune status are meaningful factors for functional cure [38], our study may contribute to the monitoring of patients on ART who will be selected to participate in eradication studies. Our study suggests that in the management of HIV-1-infected patients, the recovery of CD4/CD8 ratio should be given more attention. At the same time, combined use of immune modulators that can improve CD4^+^ T-cell recovery and decrease immune activation [39] may help achieve lower HIV-1 DNA levels.

## Additional file

Additional file 1: Table S1. Factors associated with an ultralow HIV-1 DNA level (<20 copies/106 PBMCs) at week 96. Only patients with undetectable HIV-1 RNA at week 96 were analyzed. (DOCX 20 kb)

## Abbreviations

ART: Antiretroviral therapy
CI: Confidence interval
IQR: Inter-quartile range
OR: Odds ratio
PBMCs: Peripheral blood mononuclear cells

## References

[1] Deeks SG, Autran B, Berkhout B, Benkirane M, Cairns S, Chomont N (2012). Towards an HIV cure: a global scientific strategy. Nat Rev Immunol.

[2] Buzon MJ, Martin-Gayo E, Pereyra F, Ouyang Z, Sun H, Li JZ (2014). Long-term antiretroviral treatment initiated at primary HIV-1 infection affects the size, composition, and decay kinetics of the reservoir of HIV-1-infected CD4 T cells. J Virol.

[3] Wong JK, Hezareh M, Gunthard HF, Havlir DV, Ignacio CC, Spina CA (1997). Recovery of replication-competent HIV despite prolonged suppression of plasma viremia. Science.

[4] Finzi D, Hermankova M, Pierson T, Carruth LM, Buck C, Chaisson RE (1997). Identification of a reservoir for HIV-1 in patients on highly active antiretroviral therapy. Science.

[5] Chun TW, Stuyver L, Mizell SB, Ehler LA, Mican JA, Baseler M (1997). Presence of an inducible HIV-1 latent reservoir during highly active antiretroviral therapy. Proc Natl Acad Sci U S A.

[6] Barton K, Winckelmann A, Palmer S (2016). HIV-1 reservoirs during suppressive therapy. Trends Microbiol.

[7] Williams JP, Hurst J, Stohr W, Robinson N, Brown H, Fisher M (2014). HIV-1 DNA predicts disease progression and post-treatment virological control. elife.

[8] Li JZ, Etemad B, Ahmed H, Aga E, Bosch RJ, Mellors JW (2016). The size of the expressed HIV reservoir predicts timing of viral rebound after treatment interruption. AIDS.

[9] Rouzioux C, Hubert JB, Burgard M, Deveau C, Goujard C, Bary M (2005). Early levels of HIV-1 DNA in peripheral blood mononuclear cells are predictive of disease progression independently of HIV-1 RNA levels and CD4+ T cell counts. J Infect Dis.

[10] Gianotti N, Canducci F, Galli L, Cossarini F, Salpietro S, Poli A*,* et al. HIV DNA loads, plasma residual viraemia and risk of virological rebound in heavily treated, virologically suppressed HIV-infected patients. Clin Microbiol Infect. 2015;21(1):103 e7- e10. 10.1016/j.cmi.2014.08.004 25636935

[11] Burgard M, Boufassa F, Viard JP, Garrigue I, Ruffault A, Izopet J (2009). Factors influencing peripheral blood mononuclear cell-associated HIV-1 DNA level after long-term suppressive antiretroviral therapy in 236 patients. AIDS.

[12] Boulassel MR, Chomont N, Pai NP, Gilmore N, Sekaly RP, Routy JP (2012). CD4 T cell nadir independently predicts the magnitude of the HIV reservoir after prolonged suppressive antiretroviral therapy. J Clin Virol.

[13] Watanabe D, Ibe S, Uehira T, Minami R, Sasakawa A, Yajima K (2011). Cellular HIV-1 DNA levels in patients receiving antiretroviral therapy strongly correlate with therapy initiation timing but not with therapy duration. BMC Infect Dis.

[14] Hocqueloux L, Avettand-Fenoel V, Jacquot S, Prazuck T, Legac E, Melard A (2013). Long-term antiretroviral therapy initiated during primary HIV-1 infection is key to achieving both low HIV reservoirs and normal T cell counts. J Antimicrob Chemother.

[15] Sarmati L, Parisi SG, Nicastri E, d'Ettorre G, Palmisano L, Andreotti M (2005). Association between cellular human immunodeficiency virus DNA level and immunological parameters in patients with undetectable plasma viremia level during highly active antiretroviral therapy. J Clin Microbiol.

[16] Hatano H, Jain V, Hunt PW, Lee TH, Sinclair E, Do TD (2013). Cell-based measures of viral persistence are associated with immune activation and programmed cell death protein 1 (PD-1)-expressing CD4+ T cells. J Infect Dis.

[17] Ruggiero A, De Spiegelaere W, Cozzi-Lepri A, Kiselinova M, Pollakis G, Beloukas A (2015). During stably suppressive antiretroviral therapy integrated HIV-1 DNA load in peripheral blood is associated with the frequency of CD8 cells expressing HLA-DR/DP/DQ. EBioMedicine.

[18] Persaud D, Gay H, Ziemniak C, Chen YH, Piatak M, Chun TW (2013). Absence of detectable HIV-1 viremia after treatment cessation in an infant. N Engl J Med.

[19] Saez-Cirion A, Bacchus C, Hocqueloux L, Avettand-Fenoel V, Girault I, Lecuroux C (2013). Post-treatment HIV-1 controllers with a long-term virological remission after the interruption of early initiated antiretroviral therapy ANRS VISCONTI study. PLoS Pathog.

[20] Li T, Guo F, Li Y, Zhang C, Han Y, Lye W (2014). An antiretroviral regimen containing 6 months of stavudine followed by long-term zidovudine for first-line HIV therapy is optimal in resource-limited settings: a prospective, multicenter study in China. Chin Med J.

[21] Xie J, Han Y, Qiu Z, Li Y, Li Y, Song X (2016). Prevalence of hepatitis B and C viruses in HIV-positive patients in China: a cross-sectional study. J Int AIDS Soc.

[22] Cartwright EK, Spicer L, Smith SA, Lee D, Fast R, Paganini S (2016). CD8(+) lymphocytes are required for maintaining viral suppression in SIV-infected macaques treated with short-term antiretroviral therapy. Immunity.

[23] Cao W, Mehraj V, Kaufmann DE, Li T, Routy J-P. Elevation and persistence of CD8 T-cells in HIV infection: the Achilles heel in the ART era. J Int AIDS Soc. 2016;19(1) 10.7448/IAS.19.1.20697 PMC4779330 26945343

[24] Chun TW, Justement JS, Pandya P, Hallahan CW, McLaughlin M, Liu S (2002). Relationship between the size of the human immunodeficiency virus type 1 (HIV-1) reservoir in peripheral blood CD4+ T cells and CD4+:CD8+ T cell ratios in aviremic HIV-1-infected individuals receiving long-term highly active antiretroviral therapy. J Infect Dis.

[25] Gandhi RT, McMahon DK, Bosch RJ, Lalama CM, Cyktor JC, Macatangay BJ (2017). Levels of HIV-1 persistence on antiretroviral therapy are not associated with markers of inflammation or activation. PLoS Pathog.

[26] Taylor JM, Fahey JL, Detels R, Giorgi JV (1989). CD4 percentage, CD4 number, and CD4:CD8 ratio in HIV infection: which to choose and how to use. J Acquir Immune Defic Syndr.

[27] Serrano-Villar S, Gutierrez C, Vallejo A, Hernandez-Novoa B, Diaz L, Abad Fernandez M (2013). The CD4/CD8 ratio in HIV-infected subjects is independently associated with T-cell activation despite long-term viral suppression. J Inf Secur.

[28] Serrano-Villar S, Perez-Elias MJ, Dronda F, Casado JL, Moreno A, Royuela A (2014). Increased risk of serious non-AIDS-related events in HIV-infected subjects on antiretroviral therapy associated with a low CD4/CD8 ratio. PLoS One.

[29] Serrano-Villar S, Sainz T, Lee SA, Hunt PW, Sinclair E, Shacklett BL (2014). HIV-infected individuals with low CD4/CD8 ratio despite effective antiretroviral therapy exhibit altered T cell subsets, heightened CD8+ T cell activation, and increased risk of non-AIDS morbidity and mortality. PLoS Pathog.

[30] Mudd JC, Lederman MM (2014). CD8 T cell persistence in treated HIV infection. Curr Opin HIV AIDS.

[31] Mussini C, Lorenzini P, Cozzi-Lepri A, Lapadula G, Marchetti G, Nicastri E (2015). CD4/CD8 ratio normalisation and non-AIDS-related events in individuals with HIV who achieve viral load suppression with antiretroviral therapy: an observational cohort study. Lancet HIV.

[32] Cockerham LR, Siliciano JD, Sinclair E, O'Doherty U, Palmer S, Yukl SA (2014). CD4+ and CD8+ T cell activation are associated with HIV DNA in resting CD4+ T cells. PLoS One.

[33] Murray JM, Zaunders JJ, McBride KL, Xu Y, Bailey M, Suzuki K (2014). HIV DNA subspecies persist in both activated and resting memory CD4+ T cells during antiretroviral therapy. J Virol.

[34] Chomont N, El-Far M, Ancuta P, Trautmann L, Procopio FA, Yassine-Diab B (2009). HIV reservoir size and persistence are driven by T cell survival and homeostatic proliferation. Nat Med.

[35] Barouch DH, Deeks SG (2014). Immunologic strategies for HIV-1 remission and eradication. Science.

[36] North TW, Higgins J, Deere JD, Hayes TL, Villalobos A, Adamson L (2010). Viral sanctuaries during highly active antiretroviral therapy in a nonhuman primate model for AIDS. J Virol.

[37] Svicher V, Ceccherini-Silberstein F, Antinori A, Aquaro S, Perno CF, Understanding HIV (2014). Compartments and reservoirs. Curr HIV/AIDS Rep.

[38] Calin R, Hamimi C, Lambert-Niclot S, Carcelain G, Bellet J, Assoumou L (2016). Treatment interruption in chronically HIV-infected patients with an ultralow HIV reservoir. AIDS.

[39] Li T, Xie J, Li Y, Routy JP, Li Y, Han Y (2015). Tripterygium wilfordii hook F extract in cART-treated HIV patients with poor immune response: a pilot study to assess its immunomodulatory effects and safety. HIV Clin Trials.

