# Supplementary material for: A higher CD4/CD8 ratio correlates with an ultralow cell-associated HIV-1 DNA level in chronically infected patients on antiretroviral therapy: a case control study
Source: BMC Infect Dis. 2017 Dec 15;17:771. doi: 10.1186/s12879-017-2866-y (PMC5732419; doi:10.1186/s12879-017-2866-y)
Supplement: Additional file 1: Table S1. — Factors associated with an ultralow HIV-1 DNA level (<20 copies/106 PBMCs) at week 96. Only patients with undetectable HIV-1 RNA at week 96 were analyzed. (DOCX 20 kb) [file 12879_2017_2866_MOESM1_ESM.docx]

**Additional file 1: Table S1 Factors associated with an ultralow HIV-1 DNA level (<20 copies/10^6^ PBMCs) at week 96.**

| **Variables** |  | **Univariate** |  |  |  | **Multivariate** |  |
| --- | --- | --- | --- | --- | --- | --- | --- |
|  | **OR** | **95% CI** | ***P*** |  | **OR** | **95% CI** | ***P*** |
| Sex |  |  | 0.149 |  |  |  |  |
| Male | 1.00 |  |  |  |  |  |  |
| Female | 2.21 | (0.75-6.52) |  |  |  |  |  |
| Age (years) | 1.02 | (0.96-1.08) | 0.575 |  |  |  |  |
| Time from diagnosis to ART (months) | 0.98 | (0.94-1.01) | 0.199 |  |  |  |  |
| Transmission route |  |  | 0.806 |  |  |  |  |
| Homosexual | 1.00 |  |  |  |  |  |  |
| Heterosexual | 1.25 | (0.39-3.98) | 0.706 |  |  |  |  |
| Others | 0.63 | (0.06-6.80) | 0.700 |  |  |  |  |
| HIV-1 subtype |  |  | 0.948 |  |  |  |  |
| AE | 1.00 |  |  |  |  |  |  |
| B/C/BC | 1.20 | (0.29-4.94) | 0.800 |  |  |  |  |
| Others | 1.23 | (0.34-4.52) | 0.754 |  |  |  |  |
| ART therapy |  |  | 0.639 |  |  |  |  |
| AZT/d4T+3TC+NVP | 1.00 |  |  |  |  |  |  |
| TDF+3TC+EFV | 0.76 | (0.23-2.44) |  |  |  |  |  |
| Plasma viral load at enrollment (log copies/mL) | 0.81 | (0.40-1.63) | 0.556 |  |  |  |  |
| HIV-1 DNA at enrollment (log copies/10^6^ PBMCs) | 0.85 | (0.20-3.73) | 0.831 |  |  |  |  |
| CD4^+^ T cell-count at enrollment (cells/μL) | 1.00 | (1.00-1.01) | 0.161 |  |  |  |  |
| Nadir CD4^+^ T cell-count (cells/μL) | 1.00 | (1.00-1.01) | 0.151 |  |  |  |  |
| CD8^+^ T cell-count at enrollment (cells/μL) | 1.00 | (1.00-1.00) | 0.543 |  |  |  |  |
| CD4/CD8 ratio at enrollment (per 0.1 increase) | 1.17 | (0.94-1.46) | 0.161 |  |  |  |  |
| CD4^+^ T-cell count at week 96 (cells/μL) | 1.00 | (1.00-1.01) | 0.743 |  |  |  |  |
| CD8^+^ T-cell count at week 96 (cells/μL) | 0.99# | (0.99-1.00) | 0.029 |  | 1.00 | (0.99-1.00) | 0.410 |
| CD4/CD8 ratio at week 96 (per 0.1 increase) | 1.26 | (1.04-1.53) | 0.021 |  | 1.26 | (1.04-1.53) | 0.021 |

Only patients with undetectable HIV-1 RNA at week 96 were analyzed.

OR, odds ratio, CI, confidence interval; ART, antiretroviral therapy; PBMCs, peripheral blood mononuclear cells.

0.99#, 0.996.
